# Supplementary material for: The genome of a subterrestrial nematode reveals adaptations to heat
Source: Nat Commun. 2019 Nov 21;10:5268. doi: 10.1038/s41467-019-13245-8 (PMC6872716; doi:10.1038/s41467-019-13245-8)
Supplement: Supplementary file 1 — Supplementary Information [file 41467_2019_13245_MOESM1_ESM.pdf]

Supplementary Information

The genome of a subterrestrial nematode reveals adaptations to heat

Weinstein et al.

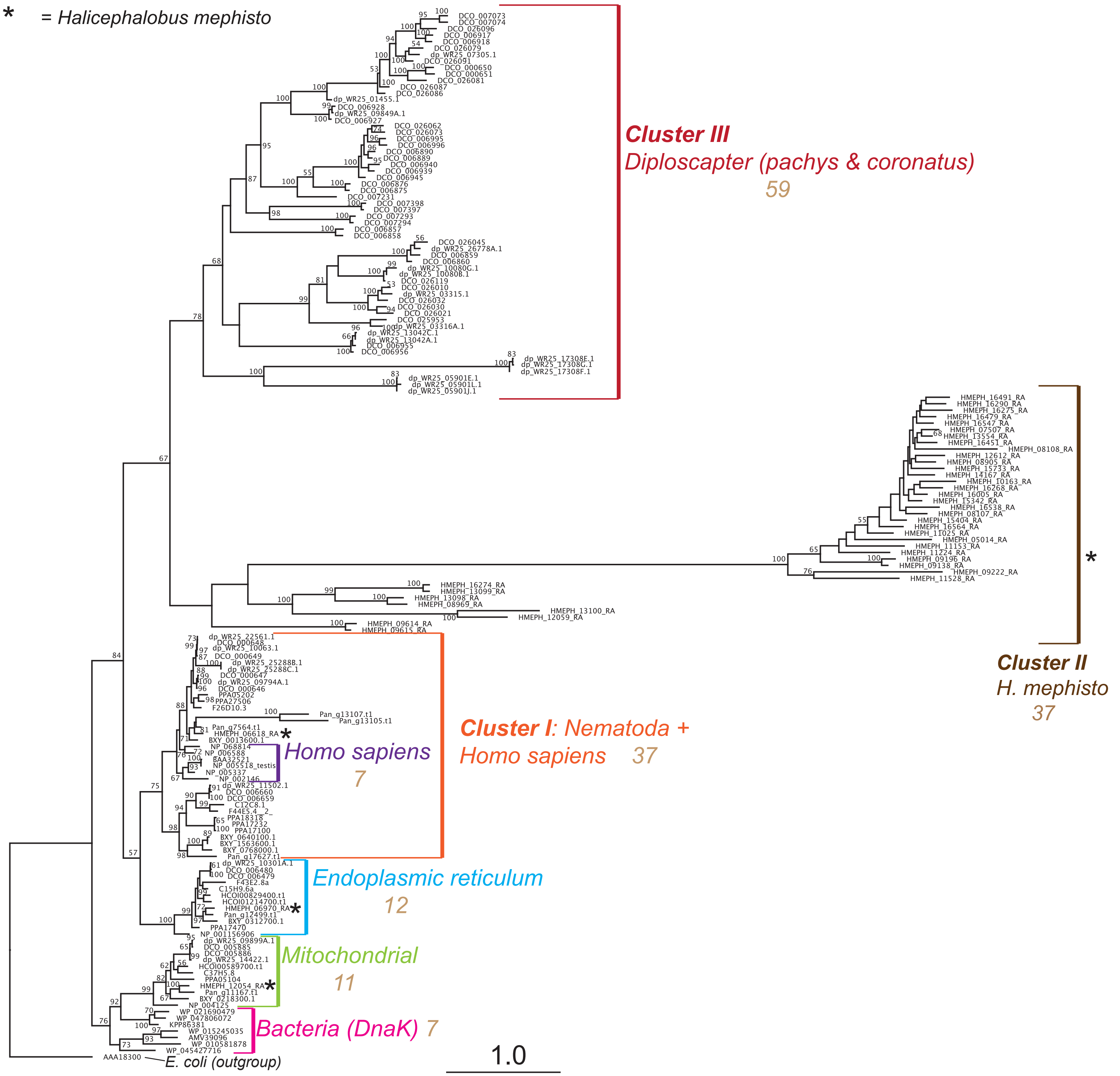

Supplementary Figure 1. RAxML phylgenetic tree of Hsp70 proteins. *H. mephisto* sequences marked with an asterisk (*). Branch numbers indicate bootstrap support, scale bar represents substitutions per site.

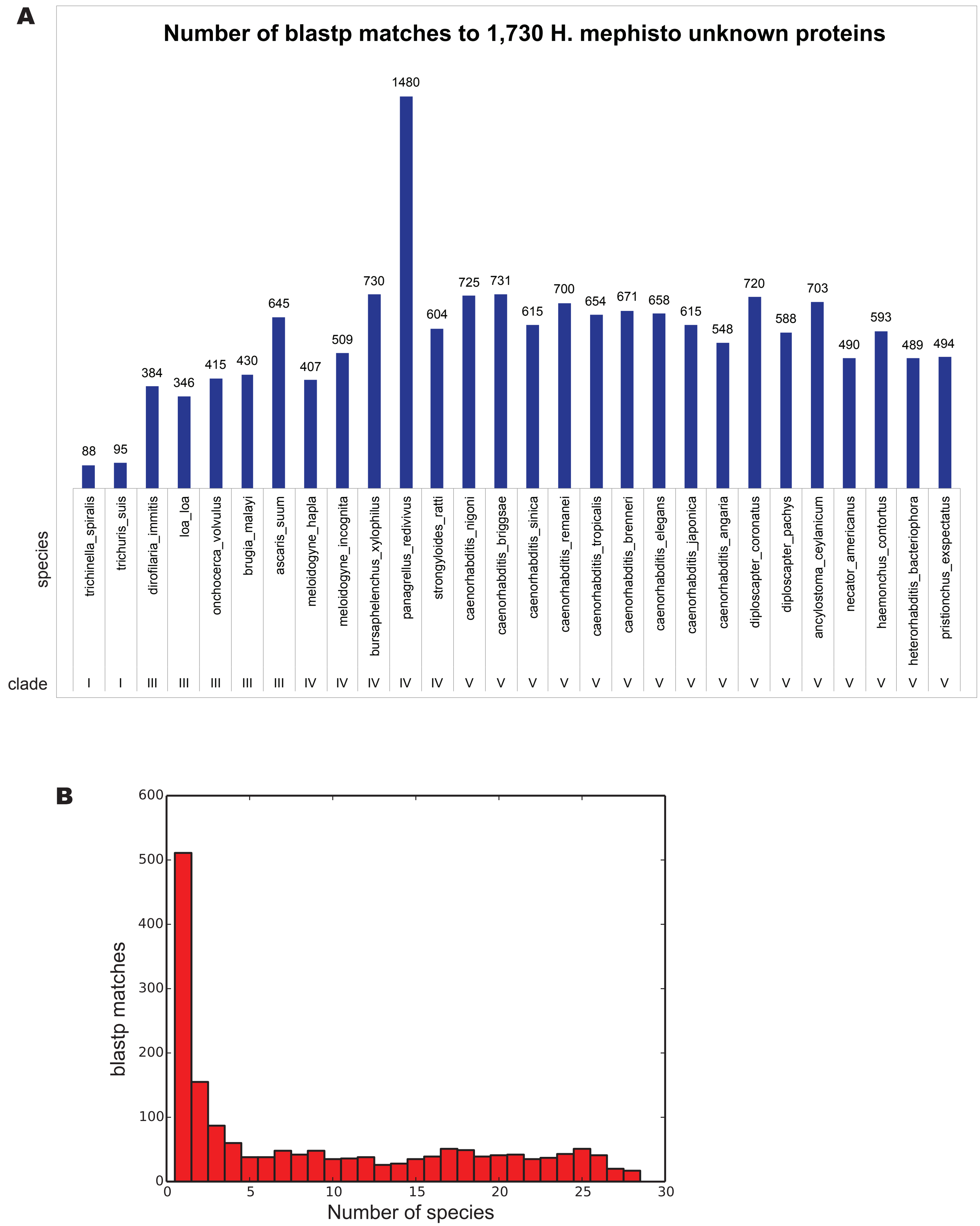

Supplementary Figure 2. A. Analysis of 1,730 *H. mephisto* genes matching to at least one nematode proteome but otherwise lacking recognizable domains by Interproscan or Pfam, and lacking matches to Uniprot-Swissprot. The evalue threshold was set to 1e-4 and number of matches per species are plotted. B. Histogram showing the number of species matched for each of the 1,730 intra-nematode proteins.

Supplementary Figure 3

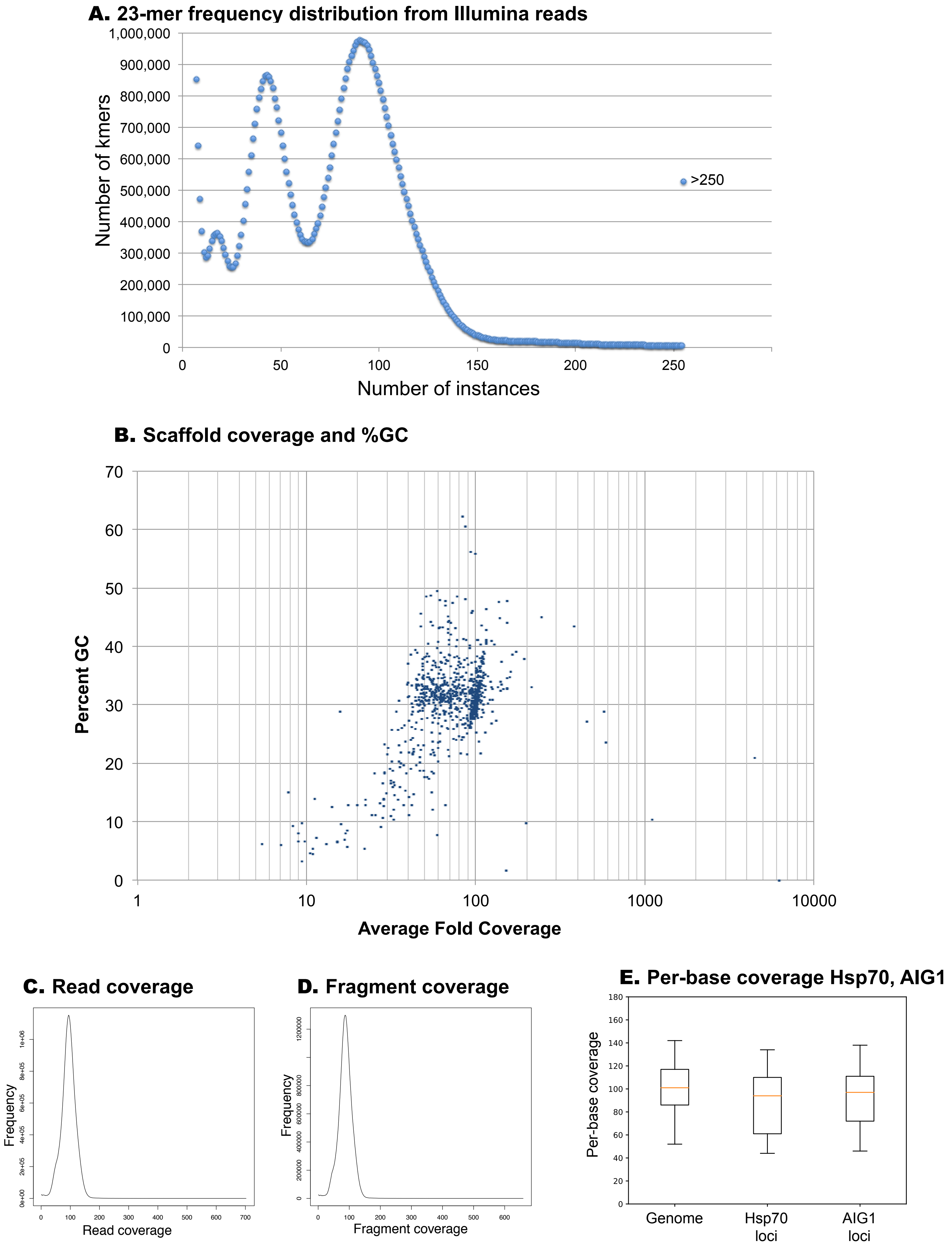

Supplementary Figure 3. K-mer frequency and assembly coverage. A. Kmer frequency distribution (23-mers). B. Per scaffold coverage vs percent GC. C. Assembled read coverage from CEGMA. D. Assembled fragment coverage from CEGMA. E. Boxplot of per-base coverage of the *H. mephisto* genome, Hsp70 loci, and AIG1 loci.

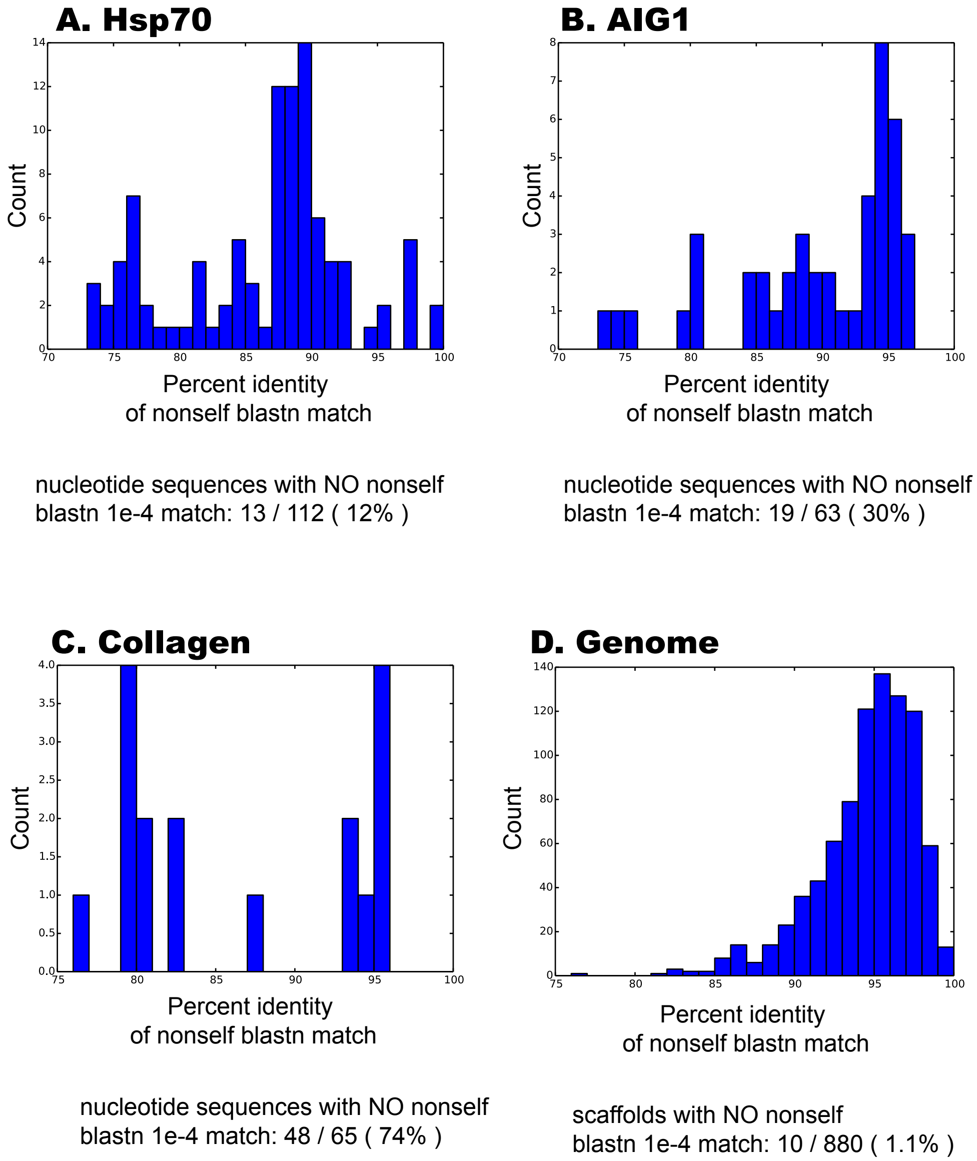

Supplementary Figure 4. Analysis of degree of similarity at nucleotide level between members of expanded gene families. Self-self blastn was performed with evalue 1e-4, then the percent identity of the first non-self match was plotted. A. Hsp70, B, AIG1, C, Collagen (control), D, full genome (control).

Supplementary Table 1. Accession numbers and fasta files used in genome annotation and blast analysis.

**Species**	**Accession**	**Fasta File**
ancylostoma_ceylanicum	ancylostoma_ceylanicum_PRJNA231479	ancylostoma_ceylanicum.PRJNA231479.WBPS12.protein.fa
ascaris_suum	ascaris_suum_PRJNA62057	ascaris_suum.PRJNA62057.WBPS12.protein.fa
brugia_malayi	brugia_malayi_PRJNA10729	brugia_malayi.PRJNA10729.WBPS12.protein.fa
bursaphelenchus_xylophilus	bursaphelenchus_xylophilus_PRJEA64437	bursaphelenchus_xylophilus.PRJEA64437.WBPS12.protein.fa
caenorhabditis_angaria	caenorhabditis_angaria_PRJNA51225	caenorhabditis_angaria.PRJNA51225.WBPS12.protein.fa
caenorhabditis_brenneri	caenorhabditis_brenneri_PRJNA20035	caenorhabditis_brenneri.PRJNA20035.WBPS12.protein.fa
caenorhabditis_briggsae	caenorhabditis_briggsae_PRJNA10731	caenorhabditis_briggsae.PRJNA10731.WBPS12.protein.fa
caenorhabditis_elegans	caenorhabditis_elegans_PRJNA13758	caenorhabditis_elegans.PRJNA13758.WBPS12.protein.fa
caenorhabditis_japonica	caenorhabditis_japonica_PRJNA12591	caenorhabditis_japonica.PRJNA12591.WBPS12.protein.fa
caenorhabditis_nigoni	caenorhabditis_nigoni_PRJNA384657	caenorhabditis_nigoni.PRJNA384657.WBPS12.protein.fa
caenorhabditis_remanei	caenorhabditis_remanei_PRJNA248909	caenorhabditis_remanei.PRJNA248909.WBPS12.protein.fa
caenorhabditis_sinica	caenorhabditis_sinica_PRJNA194557	caenorhabditis_sinica.PRJNA194557.WBPS12.protein.fa
caenorhabditis_tropicalis	caenorhabditis_tropicalis_PRJNA53597	caenorhabditis_tropicalis.PRJNA53597.WBPS12.protein.fa
diploscapter_coronatus	diploscapter_coronatus_PRJDB3143	diploscapter_coronatus.PRJDB3143.WBPS12.protein.fa
diploscapter_pachys	diploscapter_pachys_PRJNA280107	diploscapter_pachys.PRJNA280107.WBPS12.protein.fa
dirofilaria_immitis	dirofilaria_immitis_PRJEB1797	dirofilaria_immitis.PRJEB1797.WBPS12.protein.fa
haemonchus_contortus	haemonchus_contortus_PRJEB506	haemonchus_contortus.PRJEB506.WBPS12.protein.fa
heterorhabditis_bacteriophora	heterorhabditis_bacteriophora_PRJNA13977	heterorhabditis_bacteriophora.PRJNA13977.WBPS12.protein.fa
loa_loa	loa_loa_PRJNA246086	loa_loa.PRJNA246086.WBPS12.protein.fa
meloidogyne_hapla	meloidogyne_hapla_PRJNA29083	meloidogyne_hapla.PRJNA29083.WBPS12.protein.fa
meloidogyne_incognita	meloidogyne_incognita_PRJEB8714	meloidogyne_incognita.PRJEB8714.WBPS12.protein.fa
necator_americanus	necator_americanus_PRJNA72135	necator_americanus.PRJNA72135.WBPS12.protein.fa
onchocerca_volvulus	onchocerca_volvulus_PRJEB513	onchocerca_volvulus.PRJEB513.WBPS12.protein.fa
panagrellus_redivivus	panagrellus_redivivus_PRJNA186477	panagrellus_redivivus.PRJNA186477.WBPS12.protein.fa
pristionchus_exspectatus	pristionchus_exspectatus_PRJEB6009	pristionchus_exspectatus.PRJEB6009.WBPS12.protein.fa
strongyloides_ratti	strongyloides_ratti_PRJEB125	strongyloides_ratti.PRJEB125.WBPS12.protein.fa
trichinella_spiralis	trichinella_spiralis_PRJNA12603	trichinella_spiralis.PRJNA12603.WBPS12.protein.fa
trichuris_suis	trichuris_suis_PRJNA179528	trichuris_suis.PRJNA179528.WBPS12.protein.fa
