# Supplementary material for: The genome of a subterrestrial nematode reveals adaptations to heat
Source: Nat Commun. 2019 Nov 21;10:5268. doi: 10.1038/s41467-019-13245-8 (PMC6872716; doi:10.1038/s41467-019-13245-8)
Supplement: Supplementary file 2 — Reporting Summary [file 41467_2019_13245_MOESM2_ESM.pdf]

nature research

Corresponding author(s): John Bracht

Last updated by author(s): Oct 11, 2019

# Reporting Summary

Nature Research wishes to improve the reproducibility of the work that we publish. This form provides structure for consistency and transparency in reporting. For further information on Nature Research policies, see Authors & Referees and the Editorial Policy Checklist .

## Statistics

For all statistical analyses, confirm that the following items are present in the figure legend, table legend, main text, or Methods section.

| n/a | Confirmed | |
|---|---|---|
| ☐ | ☒ | The exact sample size ($n$) for each experimental group/condition, given as a discrete number and unit of measurement |
| ☐ | ☒ | A statement on whether measurements were taken from distinct samples or whether the same sample was measured repeatedly |
| ☐ | ☒ | The statistical test(s) used AND whether they are one- or two-sided<br>*Only common tests should be described solely by name; describe more complex techniques in the Methods section.* |
| ☒ | ☐ | A description of all covariates tested |
| ☐ | ☒ | A description of any assumptions or corrections, such as tests of normality and adjustment for multiple comparisons |
| ☐ | ☒ | A full description of the statistical parameters including central tendency (e.g. means) or other basic estimates (e.g. regression coefficient) AND variation (e.g. standard deviation) or associated estimates of uncertainty (e.g. confidence intervals) |
| ☐ | ☒ | For null hypothesis testing, the test statistic (e.g. $F$, $t$, $r$) with confidence intervals, effect sizes, degrees of freedom and $P$ value noted<br>*Give P values as exact values whenever suitable.* |
| ☐ | ☒ | For Bayesian analysis, information on the choice of priors and Markov chain Monte Carlo settings |
| ☒ | ☐ | For hierarchical and complex designs, identification of the appropriate level for tests and full reporting of outcomes |
| ☒ | ☐ | Estimates of effect sizes (e.g. Cohen's $d$, Pearson's $r$), indicating how they were calculated |

*Our web collection on statistics for biologists contains articles on many of the points above.*

## Software and code

Policy information about availability of computer code

| | |
|---|---|
| Data collection | no software was used to collect data. |
| Data analysis | Open source software used, and downloaded from public repositories: Python 2.7, Platanus v. 1.2.4, SoapEC v. 1.01 (from SOAPdenovo2), PBJelly v. 15.8.24, REAPR v. 1.0.18, BWA v. 0.7.12, Repeat Modeler 1.0.11, RepeatMasker v 4.0.6 , Hmmer 3.1.b2, Maker 3.01.1, TopHat 2.1.1, Stringtie v 1.3.4, Ballgown 2.12.0, TransDecoder 5.3.0, OrthoMCL v2.0.9, orthAgogue v1.0.3, MUSCLE v3.8.3.1, ProtTest v3.4.2, raxml-ng v0.4.1b, GOATools v0.6.10, Interproscan v5.30.69, MAFFT v7.017, MrBayes 2.2.4, RAxML v8.2.12, PAML v. 4.9 |

For manuscripts utilizing custom algorithms or software that are central to the research but not yet described in published literature, software must be made available to editors/reviewers. We strongly encourage code deposition in a community repository (e.g. GitHub). See the Nature Research guidelines for submitting code & software for further information.

## Data

Policy information about availability of data

All manuscripts must include a data availability statement. This statement should provide the following information, where applicable:

- Accession codes, unique identifiers, or web links for publicly available datasets
- A list of figures that have associated raw data
- A description of any restrictions on data availability

This Whole Genome Shotgun project has been deposited at DDBJ/ENA/GenBank under the accession SWDT00000000. The version described in this paper is version SWDT01000000. The raw Illumina DNA and RNA data, and PacBio DNA data are available on the Sequence Reads Archive (SRA) at accession PRJNA528747. Transcriptome expression data as FPKM values genome-wide is available in the Gene Expression Omnibus (GEO) at accession GSE133178. The genome annotation file and transcript and protein predictions are available from WormBase Parasite at accession Halicephalobus_mephisto_prjna528747. The source data underlying Figures 4A and 4B are provided as a Source Data File.

# Field-specific reporting

Please select the one below that is the best fit for your research. If you are not sure, read the appropriate sections before making your selection.

[x] Life sciences [ ] Behavioural & social sciences [ ] Ecological, evolutionary & environmental sciences

For a reference copy of the document with all sections, see nature.com/documents/nr-reporting-summary-flat.pdf

# Life sciences study design

All studies must disclose on these points even when the disclosure is negative.

| | |
|---|---|
| Sample size | One DNA sample sequenced twice, one by Illumina and once by PacBio, and 12 RNA samples sequenced. For DNA, n=1 and for RNA n=12. |
| Data exclusions | No data were excluded from analysis. |
| Replication | This is a genomic study with n=12 transcriptomes, which were all folded into the analysis. |
| Randomization | n/a |
| Blinding | n/a |

# Reporting for specific materials, systems and methods

We require information from authors about some types of materials, experimental systems and methods used in many studies. Here, indicate whether each material, system or method listed is relevant to your study. If you are not sure if a list item applies to your research, read the appropriate section before selecting a response.

## Materials & experimental systems

| n/a | Involved in the study | |
|---|---|---|
| [x] | [ ] | Antibodies |
| [x] | [ ] | Eukaryotic cell lines |
| [x] | [ ] | Palaeontology |
| [ ] | [x] | Animals and other organisms |
| [x] | [ ] | Human research participants |
| [x] | [ ] | Clinical data |

## Methods

| n/a | Involved in the study | |
|---|---|---|
| [x] | [ ] | ChIP-seq |
| [x] | [ ] | Flow cytometry |
| [x] | [ ] | MRI-based neuroimaging |

## Animals and other organisms

Policy information about studies involving animals; ARRIVE guidelines recommended for reporting animal research

| | |
|---|---|
| Laboratory animals | We used the nematode Halicephalobus mephisto. |
| Wild animals | These animals were obtained by filtering water obtained from a gold mine in South Africa, and were then cultured in the laboratory and collected for nucleic acids. |
| Field-collected samples | Worms are cultured identically to C. elegans in the laboratory, and can be grown on the same food and same plates. |
| Ethics oversight | Since no vertebrates were used in the study no ethical oversight was required for the study. |

Note that full information on the approval of the study protocol must also be provided in the manuscript.
